# Association between dental clinical measures and oral health-related quality of life among Iraqi schoolchildren: A cross-sectional study

**DOI:** 10.1371/journal.pone.0293024

**Published:** 2024-04-25

**Authors:** Hanan Fadhil Alautry, Mahshid Namdari, Mohammad Hossein Khoshnevisan, Hadi Ghasemi

**Affiliations:** 1 Department of Community Oral Health, School of Dentistry, Shahid Beheshti University of Medical Sciences, Tehran, Iran; 2 Department of Biostatistics, School of Allied Medical Sciences, Shahid Beheshti University of Medical Sciences, Tehran, Iran; University of Puthisastra, CAMBODIA

## Abstract

**Objective:**

The aim of this study was to assess the association between dental clinical measures and oral health-related quality of life (OHRQoL) together with the potential mediating role of sociodemographic factors and oral health behaviours on this association in a group of Iraqi children.

**Methods:**

The target population for this cross-sectional study consisted of 372 primary school children aged 8–10 years in the city of Kut, Iraq, during the year 2022. The participants were selected using a multi-stage random sampling technique. Detailed information about the children was collected through a comprehensive questionnaire that included demographic characteristics, oral health-related behaviors, the Arabic version of the Child Perceptions Questionnaire for 8-10-year-olds (CPQ_8-10_), and parental knowledge regarding oral health. Additionally, clinical dental evaluations were conducted, which included assessments of decayed, missing, and filled surfaces (DMFS, dmfs) as well as teeth (DMFT, dmft). Simplified oral hygiene index (OHI-S), bleeding on probing (BOP), and the community periodontal index (CPI) were also recorded for each participant using the recommended methodology by the World Health Organization (WHO). The statistical analysis included the Chi-square test, independent t-test, and simple and multiple linear regressions.

**Results:**

The mean age of the children was 9.0 (± 0.82) years. About one-third of children reported brushing at least two times per day and consuming a sweet snack once a day. Visiting a dentist during the past year was reported by 21% of children. Oral health behaviours demonstrated a significant association with the total CPQ_8-10_ scores (p < 0.001). Based on adjusted effects (β and 95% CI) from the multiple linear regressions, untreated dental caries (dt > 0, DT > 0) had a negative impact on the total CPQ_8-10_ score (adjusted β = 2.3 (95% CI: 0.67 to 3.91) and 3.4 (95% CI: 2.14 to 4.56), respectively). Decayed surfaces (DS), and teeth (DT) were associated with the total score of the CPQ_8-10_ and all its subscales (adjusted β range = 0.1 (95% C.I.: 0.03 to 0.19)–1.0 (95% CI: 0.72 to 1.26) and 0.2 (95% CI: 0.004 to 0.40)–1.2 (95% CI: 0.91 to 1.67), respectively). There was an association between oral hygiene index and total CPQ_8-10_ scores (adjusted β = 1.8 (95% CI: 0.62 to 3.02)), especially the functional limitations and emotional well-being subscales.

**Conclusion:**

Findings of this study emphasizes the negative impact of dental caries and poor oral hygiene on children’s OHRQoL. This association is highlighted more when considering that over two-thirds of these children do not comply with favourable levels of oral health behaviour. Enhancing the level of OHRQoL among these children, therefore, necessitates comprehensive programs for decreasing the volume of unmet oral health needs and improving children’s adherence to recommended oral health behaviours.

## Introduction

Oral health-related quality of life (OHRQoL) has been used extensively as a measure of the impact for oral disorders on individuals and societies. This measure aims to capture the broad consequences of various levels of oral health from the perspective of affected people [1,2]. Moreover, it can serve as an outcome measure for identifying patient concerns regarding the main functions of the mouth and dentition after oral health interventions [2]. The measurement of OHRQoL provides fundamental information for oral health needs assessments of individuals and communities, clinical decision-making, and the estimation of the need for future services in public health programmes [3–6].

Research on OHRQoL has demonstrated substantial implications for children’s health in terms of both clinical and public health aspects of dentistry. Untreated dental decay, which is prevalent among children in different parts of the world [7], causes pain, and infection as well as negatively influences eating, sleeping, studying, and the psychosocial aspects of children’s lives. Consequently, some countries have included the assessment of OHRQoL as part of their national oral health surveys [8,9].

Several studies have provided evidence of the correlation between OHRQoL and dental caries, as well as periodontal diseases in various populations and different age groups [10–17]. Considering that dental caries and gum diseases affect about 80% of children worldwide [18], it is crucial to gather information on the impact of oral health on the quality of life of schoolchildren. This is because the health status during childhood ultimately determines the health status in later stages of life [19]. Moreover, demographic characteristics and dental clinical measures exhibit varying influences on the OHRQoL in children [20]. Therefore, in order to enhance the OHRQoL, oral health promotion strategies should take into account the existing oral health status of children and its relationship with their social and environmental factors. It has been estimated that 66% of primary schoolchildren in Iraq are afflicted with dental caries [21], a figure that falls short of the World Health Organization’s target of 50% caries-free children set in the year 2000 [22]. Detailed insights regarding the impact of this disease on the well-being of Iraqi children are limited. As a result, the primary objective of this investigation was to evaluate the correlation between dental clinical measures and OHRQoL in a group of 8-10-year-old children from Iraq. Furthermore, we explored the potential mediating influence of sociodemographic factors and oral health behaviours on the aforementioned correlation.

## Materials and methods

The target population for the present cross-sectional investigation consisted of children aged 8 to 10 years who attended elementary schools in Kut City, Iraq. These children were chosen through a multistage random sampling process. The sample size for the study was determined using G*power software version 3.1.9.7 [23]. With the assumption of 17 predictors in the regression model and an anticipated effect size of 0.15 [24], a power of 0.90, and α = 0.05, a total of 179 samples were deemed necessary. Taking into account the cluster sampling method and allowing for a potential 20% of incomplete responses, the total sample size calculation amounted to 372 individuals. This number was then evenly distributed based on the gender and age of the children, resulting in 62 children from each age group (8, 9, and 10 years) with an equal number of boys and girls included in the final sample population. The inclusion criteria for participation in the study were Iraqi nationality and an age range of 8 to 10 years. The exclusion criteria encompassed any systemic illness, disabilities, and failure to provide consent for participation in the study. To identify the study subjects, two girls’ and two boys’ schools were randomly selected from a list of all 40 elementary public schools in the city.

Data for the present study was gathered by means of a questionnaire with the following sections: 1) demographic characteristics of the students including age, gender, parents’ age and level of education. 2) questions related to the students’ oral health-related behaviour such as brushing frequency, eating sugary snacks, and last dental visit. 3) the Arabic version of the CPQ_8-10_ questionnaire, and 4) questions regarding parental knowledge of oral health and caries preventive measures. The CPQ_8-10_ composed of 25 items distributed among four subscales: oral symptoms (five items), functional limitations (five items), emotional well-being (five items), and social well-being (10 items). The items were related to events that occurred in the four weeks prior to the completion of the questionnaire. Each item was followed by answer options on a 5-point Likert scale (never, once or twice, sometimes, often, and every day or almost every day). Each option was scored from 0–4 and summed up to obtain a total score with a theoretical range of 0–100, in which higher scores denoted poorer OHRQoL. The last section of the questionnaire included 20 statements regarding various aspects of parental knowledge of oral health and caries preventive measures, which were originally adopted from the questionnaires of two previous studies [25,26]. For this section, the parents were expected to reflect their ideas about each item on a 3-point Likert scale (disagree, do not know, and agree, which were scored as 0, 1, and 2, respectively). The sum of the scores for all items, with a theoretical range of 0–40, constituted respondents’ knowledge, with higher scores denoting a higher level of knowledge. Data collection also included clinical dental examination based on WHO-recommended method [27], in which indices decayed, missing and filled surfaces (DMFS, dmfs) and teeth (DMFT, dmft), the simplified oral hygiene index (OHI-S), bleeding on probing (BOP), and the community periodontal index (CPI) [28] were recorded for each of the participating schoolchildren.

The Ethics Committee of the Shahid Beheshti School of dentistry approved this study (IR.SBMU.DRC.REC.1401.030). Permission was also obtained from the General Directorate of Education of Kut City and the selected school principals. In each class, the purpose of the study was explained to the students by one of the authors (HA). Then the questionnaire and the written consent form were distributed to the students for taking them home. The students were asked to pass the questionnaire and consent form to their parents in order to be completed, signed and returned to the schools in the subsequent days. Explanation was provided to children and their parents by HA in case of any question regarding the content of the questionnaire. The clinical oral examination was conducted by two experienced dentists (HF and a colleague). Before the main data collection, a calibration program was performed in which two examiners assessed caries experience, oral hygiene, and periodontal status on a group of 20 children (10 boys and 10 girls) selected from the designated schools. The calibration procedure encompassed a demonstration of the examination sheet and questionnaires, which lasted for two hours, facilitated by one of the authors (HA). Subsequently, two examiners administered the oral examination and completed the questionnaires twice with a two-week interval for each of the 20 children. Notably, the data derived from these children was not incorporated into the main study. The kappa coefficients for inter-examiner and intra-examiner agreement were > 0.8 for all dental clinical measures evaluated.

The recruitment period lasted from October to December 2022, on each working day, a group of five children was brought to the examination room provided by the school authority. After the signed consent and completed questionnaires were checked, oral examination was conducted using WHO probes (Dentirak®) with a 0.5 mm ball end and disposable mouth mirrors with the child seated on a mobile dental chair using artificial dental unit light.

The validity and reliability of the Arabic version of CPQ_8-10_ have been confirmed previously [29]. To confirm the validity and reliability of parental knowledge questionnaire, the following steps were taken. First, the English version was translated into Arabic using the forwards-backwards technique [30]. Second, the content validity index (CVI) and content validity ratio (CVR) of all items were calculated by asking eight experts from departments of paediatric dentistry and dental public health to rate their opinion about the necessity, simplicity, clarity, and relevance of each item [31]. This resulted in CVI = 0.94 and CVR = 0.92, which showed an acceptable level of validity. Third, to determine the reliability of this section of the questionnaire, parents of 20 schoolchildren were asked to complete the questionnaire twice with a two-week interval using the test-retest method. This process resulted in an intraclass correlation coefficient (ICC) of 0.83, which was in the acceptable range.

The chi-square test and independent t-test were used for comparisons between variables based on gender. Simple linear regressions were used to examine the association between the dental clinical measures, demographic and oral health-related behaviour variables with the CPQ_8-10_ scores. Multiple linear regression analyses were conducted to determine the adjusted effect of dental clinical measures on CPQ_8-10_ scores by adding covariates (oral health-related behaviours, parents’ knowledge and sociodemographic variables), which were found to be significant at P < 0.25 in the simple linear regression analysis [32]. The data were analysed using the IBM SPSS program version 25 (Armonk®, NY).

## Results

A total of 372 children aged 8–10 years (51% boys) constituted the study population. Distribution of the children according to their background factors and frequency of oral health behaviours is presented in Table 1. The children’s age was evenly distributed among 8, 9, and 10 years, their mother were mostly younger than 40 years, and educational level of more than half of their parents were graduate. Mean scores for parental knowledge about prevention of oral diseases was 33 out of the range 0–40. Around one third of the children reported toothbrushing at least twice per day (more girls than boys; p = 0.002) and ≤ once per day sweet snacking between meals. About one out of five children reported to have a dental visit during last year.

**Table 1:** Distribution of the children (n = 372) based on their sociodemographic factors and oral health behaviours.

Variables		All n (%)	Boys n (%)	Girls n (%)		p value ^a^
Age (years)	8	122 (33%)	58 (30%)	64 (35%)		0.237
	9	119 (32%)	58 (30%)	61 (34%)		
	10	131 (35%)	75 (40%)	56 (31%)		
Mother’s age (years)	< 40	242 (65%)	119 (62%)		123 (68%)	0.253
	≥ 40	130 (35%)	72 (38%)		58 (32%)	
Father’s education level	Primary school	49 (13%)	28 (15%)		21 (12%)	0.685
	Secondary or high school	123 (33%	63 (33%)		60 (33%)	
	Graduate	200 (54%)	100 (52%)		100 (55%)	
Mother’s education level	Primary school	58 (16%)	41(22%)		17 (9%)	0.001
	Secondary or high school	128 (34%)	53 (28%)		75 (41%)	
	Graduate	186 (50%)	97 (50%)		89 ((50%)	
Brushing frequency	Irregular	54 (15%)	31 (16%)		23(13%)	0.002
	Once a day	199 (53%)	115 (60%)		84 (47%)	
	≥ Twice per day	119 (32%)	45 (24%)		74 (40%)	
Sweet snacks between meals	≤ once a day	132 (36%)	71 (37%)		61 (34%)	0.484
	≥ Twice per day	240 (64%)	120 (63%)		120 (66%)	
Past dental visits	Never	152 (41%)	69 (36%)		83 (46%)	0.052
	Last year and before	140 (38%)	83 (44%)		57 (32%)	
	During last year	80 (21%)	39 (20%)		41 (22%)	
Mean of parental knowledge scores (SD)		33.0 (3.69)	33.1 (3.65)		32.9 (3.74)	0.604^b^

^a^ Statistical evaluation by the chi-square test.

^b^ Statistical evaluation by the independent sample t test.

The mean scores for dental clinical measures in the children according to their gender are presented in Table 2. On average, the DMFT index among these children was 1.5 (±1.59) with more than 90% being attributed to the D component. (girls’ higher than boys’; p = 0.01). Bleeding index was higher among girls than boys (p<0.001). Healthy periodontal status (CPI = 0) was more prevalent among boys than girls (p<0.001).

**Table 2:** Mean (SD) of dental clinical measures of the Iraqi schoolchildren (n = 372) by gender.

Clinical measures	All	Boys Girls		p value ^a^
		N = 191	N = 181	
DMFS	2.4 (3.43)	2.1 (3.29)	2.7 (3.55)	0.109
DS	2.3 (3.32)	2.1 (3.22)	2.5 (3.40)	0.196
MS	0.1 (0.51)	0.02 (0.36)	0.1 (0.64)	0.297
FS	0.1(0.44)	0.03 (0.25)	0.1 (0.58)	0.149
DMFT	1.5 (1.59)	1.3 (1.38)	1.7 (1.77)	0.009
DT	1.4 (1.57)	1.2 (1.33)	1.6 (1.76)	0.010
MT	0.01 (0.10)	0.005 (0.07)	0.01 (0.12)	0.297
FT	0.04 (0.26)	0.02 (0.19)	0.06 (0.31)	0.208
dmfs	9.5 (8.85)	9.9 (8.90)	9.1 (8.80)	0.368
ds	7.4 (7.48)	8.1 (7.92)	6.8 (6.95)	0.101
ms	1.8 (4.09)	1.6 (3.66)	2.0 (4.51)	0.440
fs	0.2 (0.94)	0.1 (0.71)	0.3 (1.14)	0.235
dmft	4.1 (2.95)	4.1 (3.01)	4.0 (2.88)	0.597
dt	3.6 (2.78)	3.8 (2.89)	3.5 (2.66)	0.285
mt	0.3 (0.82)	0.3 (0.73)	0.4 (0.91)	0.446
ft	0.07 (0.41)	0.03 (0.23)	0.1 (0.54)	0.055
Prevalence of untreated decay (%)				
DT > 0	225 (60%)	112 (59%)	113 (62%)	0.445 ^b^
dt > 0	306 (82%)	158 (83%)	148 (82%)	0.810 ^b^
OHI-S	0.9 (0.51)	0.9 (0.56)	0.9 (0.45)	0.328
BI	1.2 (1.57)	0.9 (1.18)	1.5 (1.85)	<0.001
Number (%) of children with different CPI scores				
CPI = 0	144 (38.7)	92 (48.2)	52 (28.7)	<0.001^b^
CPI = 1	190 (51.1)	81 (42.4)	109 (60.3)	
CPI = 2	38 (10.2)	18 (9.4)	20 (11.0)	

DS/ds: Decayed surfaces, MS/ms: Missing surfaces, FS/fs: Filled surfaces, DT/dt: Decayed teeth, MT/mt: Missing teeth, FT/ft: Filled teeth, DT > 0/ dt > 0, prevalence of untreated decay, OHI-S: Simplified oral hygiene index, BI: Bleeding index, CPI: Community periodontal index. CPI = 0: Healthy periodontium, CPI = 1: Bleeding only, CPI = 2: With calculus.

^a^ Statistical evaluation by the independent sample t test.

^b^ Statistical evaluation by the chi-square test.

As shown in Table 3, the mean score for the total CPQ_8-10_ was 14.7 (±7.64) most of which belonged to the domain of oral symptoms with no statistical difference based on gender.

**Table 3:** Mean (SD) of the total child perception questionnaire (CPQ_8-10_) and subscale scores of Iraqi schoolchildren (n = 372) by gender.

CPQ8-10	All	Boys Girls		p value ^a^
		N = 191	N = 181	
Total scores	14.7 (7.64)	14.8(7.48)	14.7 (7.82)	0.830
Oral symptoms	7.7(3.25)	7.5 (3.42)	7.8(3.00)	0.264
Functional limitation	3.3 (3.46)	3.6 (3.62)	3.0 (3.26)	0.119
Emotional well-being	2.3 (2.50)	2.3 (2.51)	2.4 (2.48)	0.265
Social well-being	1.4(2.85)	1.4 (2.69)	1.4 (3.01)	0.931

^a^ Statistical evaluation by independent-sample t test.

The simple linear regression for the association of CPQ_8-10_ scores with dental clinical measures is presented in Table 4. In general, dental caries, oral hygiene, and bleeding indices were all associated with CPQ_8-10_ scores. The caries measures (DMFS, dmfs) and (DMFT, dmft) were associated with the total score of the CPQ_8-10_ and all of its subscales (β range = 0.1 (95% CI: 0.05 to 0.12) –1.3 (95% CI: 1.11 to 1.49) and 0.2 (95% CI: 0.14 to 0.33) –2.2 (95% CI: 1.81 to 2.63), respectively). Additionally, there was an association between untreated decay in both primary and permanent dentition with total CPQ_8-10_ scores (β = 5.5 (95% CI: 3.53 to 7.46) and 5.8 (95% CI: 4.32 to 7.28), respectively). Poor oral hygiene was also associated with total CPQ_8-10_ scores (β = 1.8 (95% CI: 1.28 to 2.29). Regarding periodontal status, children with bleeding gum had higher CPQ_8-10_ scores (β = 2.2 (95% CI: 0.62 to 3.82)) compared to participants with healthy gum.

**Table 4:** The association between the child perception questionnaire (CPQ_8-10_) scores and dental clinical measures as assessed by simple linear regression models.

Clinical measures	Total scores-CPQ_8-10_ β (95% CI)	Oral symptoms β (95% CI)	Functional limitation β (95% CI)	Emotional well-being β (95% CI)		Social well-being β (95% CI)
DMFS	1.3(1.09,1.47)	0.2(0.08,0.27)	0.3(0.19,0.39)		0.3(0.22,0.36)	0.4(0.34,0.49)
DS	1.3(1.11,1.49)	0.2(0.08,0.28)	0.3(0.18,0.39)		0.3(0.22,0.37)	0.4(0.35,0.50)
MS	1.7 (0.88, 3.87)	0.1 (– 0.46, 0.81)	0.6 (0.06, 1.42)		0.4 (0.05, 1.04)	0.7(0.37, 1.49)
FS	0.5 (–1.22, 1.27)	0.2 (– 0.47, 1.00)	0.4 (– 0.32, 1.25)		– 0.07(– 0.64, 0.50)	– 0.1(– 0.75, 0.54)
DMFT	2.2 (1.80, 2.63)	0.3 (0.18, 0.59)	0.5 (0.33, 0.76)		0.5 (0.35, 0.66)	0.6 (0.4, 0.80)
DT	2.2 (1.81, 2.69)	0.4 (0.19, 0.61)	0.5 (0.32, 0.76)		0.5 (0.36, 0.67)	0.6 (0.45, 0.79)
MT	11.8 (4.42, 19.36)	0.8 (–2.34, 4.05)	3.7 (0.30, 7.11)		2.7 (0.29, 5.21)	4.6 (1.88, 7.45)
FT	1.5 (–1.47, 4.49)	0.4 (–0.81, 1.72)	0.8 (– 0.5, 2.17)		0.09 (–1.07, 0.88)	0.3 (– 0.72, 1.50)
DT > 0	5.8 (4.32, 7.28)	1.4 (0.83, 1.15)	1.7 (0.95, 2.36)		1.2 (0.65, 1.67)	1.2 (0.59, 1.76)
dmfs	0.5(0.40,0.55)	0.1(0.05,0.12)	0.2(0.14,0.22)		0.1(0.07,0.12)	0.1(0.08,0.14)
ds	0.5(0.44,0.62)	0.1(0.05,0.13)	0.2(0.15,0.24)		0.1(0.07,0.14)	0.1(0.09,0.16)
ms	0.4 (0.26, 0.63)	0.1(0.008, 0.16)	0.1(0.06, 0.23)		0.1 (0.02, 0.15)	0.1 (0.01, 0.16)
fs	0.03 (– 0.79, 0.85)	0.2 (– 0.07, 0.62)	–0.1 (– 0.52, 0.22)		0.1 (– 0.17, 0.36)	– 0.1(– 0.41, 0.20)
dmft	1.7 (0.93, 1.40)	0.2 (0.17, 0.39)	0.4 (0.32, 0.54)		0.2 (0.15, 0.32)	0.2 (0.14, 0.33)
dt	1.1 (0.87, 1.38)	0.2 (0.15, 0.38)	0.4 (0.32, 0.56)		0.2 (0.13, 0.31)	0.2 (0.13, 0.33)
mt	2.2 (1.33, 3.17)	0.4 (0.04, 0.84)	0.7 (0.30, 1.14)		0.4 (0.13, 0.75)	0.4 (0.11, 0.81)
ft	0.5 (–2.38, 1.35)	0.6 (– 0.15, 1.42)	0.7 (–1.57, 0.10)		0.1 (– 0.4, 0.76)	0.3 (–1.03, 0.34)
dt > 0	5.5 (3.53,7.46)	2.1 (1.28, 2.95)	2.3(1.40, 3.19)		0.8 (0.09, 1.42)	0.7 (– 0.09,1.42)
OHI	1.8(1.28,2.29)	0.4(– 0.20, 1.08)	1.0(0.35,1.72)		0.7(0.21,1.20)	0.7(0.21,1.34)
BI	0.5(0.04,1.02)	0.1 (– 0.06, 0.35)	0.1 (– 0.13, 0.30)		0.1 (– 0.10, 0.22)	0.2(0.03,0.40)
CPI scores						
CPI = 0	Reference					
CPI = 1	2.2 (0.62, 3.82)	0.9 (0.21, 1.60)	0.1 (– 0.61, 0.89)		0.2 (– 0.24, 0.84)	0.8 (0.26, 1.49)
CPI = 2	2.2 (– 0.45, 4.98)	1.1 (– 0.04, 2.26)	1.1 (– 0.06, 2.41)		0.2 (–1.09, 0.69)	0.3 (– 0.70, 1.33)

β: Regression estimate, DS/ds: Decayed surfaces, MS/ms: Missing surfaces, FS/fs: Filled surfaces, DT/dt: Decayed teeth, MT/mt: Missing teeth, FT/ft: Filled teeth, DT > 0/ dt > 0, prevalence of untreated decay, OHI-S: Simplified oral hygiene index, BI: Bleeding index, CPI: Community periodontal index, CPI = 0: Healthy periodontium, CPI = 1: Bleeding only, CPI = 2: With calculus.

Table 5 depicts the association between sociodemographic factors and oral health behaviors with CPQ_8-10_ scores, showing that parents with higher level of education were associated with lower CPQ_8-10_ scores (β = -1.4 (95% CI: -2.66 to -0.07) and -1.5 (95% CI: -2.22 to -0.80), respectively)). For oral hygiene behaviors, children with past dental visits during the last year were associated with lower CPQ_8-10_ scores (β = -6.5 (95% CI: -8.44 to -4.60)). Similarly, participants who brushed more than twice a day (β = -10 (95% CI: -11.95 to -7.88)) and once a day (β = -9.0 (95% CI: -11.21 to -6.85)) were associated with lower CPQ_8-10_, and children who ate sweet snacks between meals ≥ twice per day had higher CPQ_8-10_ scores (β = 4.8 (95% CI: 3.24 to 6.35)). Parental knowledge of oral health and caries preventive measures was associated with lower CPQ_8-10_ scores (β = -0.7 (95% CI: -0.94 to -0.55)).

**Table 5:** The association between child perception questionnaire (CPQ_8-10_) scores with oral health behaviours and sociodemographic factors as assessed by simple linear regression models.

Oral health behaviours	Total scores- CPQ_8-10_ β (95% CI)			Oral symptoms β (95% CI)	Functional limitation β (95% CI)				Emotional well-being β (95% CI)	Social well-being β (95% CI)
Brushing frequency										
Irregular		Reference								
Once a day		–9.0(–11.21,–6.85)		–0.4 (–1.49,0.55)	–2.9(–4.04,–1.93)				–2.1(–2.88,–1.36)	–3.2(–4.09,–2.42)
≥ Twice per day		–10.0(–11.95,–7.88)		–1.2(–2.16,–0.25)	–2.8(–3.85,–1.87)				–2.2(–2.91,–1.49)	–3.2(–3.99,–2.43)
Sweet snacks										
Not or once a day		Reference								
≥ Twice per day		4.8(3.24,6.35)	1.6(0.96,2.30)		1.0(0.31,1.77)				1.2(0.74,1.78)	1.1(0.55,1.74)
Past dental visit										
Never		Reference								
Last year and before		–2.9(–4.60,–1.26)	0.6 (–0.10,1.32)		–0.9(–1.72,–0.16)			–1.0(–1.69,–0.49)		–1.0(–1.82,–0.52)
During last year		–6.5(–8.44,–4.60)	–1.7(–2.62,–0.97)		–2.0(–2.96,–1.17)			–1.2(–1.94,–0.64)		–1.0(–1.85,–0.09)
Parental knowledge		–0.7(–0.94,–0.55)	–0.2(–0.33,–0.15)		–0.1(–0.25,0.06)			–0.1(–0.23,–0.10)		–0.1(–0.26,–0.10)
Sociodemographic factors										
Age (years)										
8		Reference								
9		0.1 (–1.87, 1,99)	0.1 (– 0.75, 0.89)		0.01 (– 0.86, 0.89)		– 0.2 (– 0.87, 0.39)			0.1 (– 0.59, 0.85)
10		1.0 (– 0.81, 2.96)	0.1(– 0.96, 0.64)		0.1(– 0.72, 0.99)		0.1 (–0.49, 0.74)			0.4(– 0.23, 1.17)
Gender										
Girls		Reference								
Boys		0.1 (–1.39, 1.73)	– 0.3 (–1.03, 0.28)		0.5(– 0.14, 1.26)		–0.2 (– 0.79, 0.22)			–0.02 (– 0.60, 0.55)
Mother’s age										
< 40 year		Reference								
≥ 40 year		0.2 (–1.41,1.85)	–0.6(–0.03,–1.34)		–0.1 (–1.88,0.59)		–0.01 (–0.52,0.54)			– 0.09 (–0.70, 0.51)
Mother’s education										
Primary school		Reference								
Secondary/high school		0.4 (–1.43, 2.42)	0.5 (–0.27, 1.35)			– 0.1 (– 0.98, 0.77)	– 0.3 (– 0.93, 0.33)			0.3 (– 0.40, 1.03)
Graduate		–1.4(–2.66,–0.07)	–0.4(–0.99,–0.23)			–0.3(–0.94,–0.23)	– 0.2 (– 0.65, 0.19)			– 0.2 (– 0.76, 0.20)
Father’s education										
Primary school		Reference								
Secondary/high school		–1.0 (–3.55, 1.40)	0.1 (–1.0, 1.12)			0.8 (–1.04, 1.22)	–0.5 (–1.33, 0.30)			– 0.2 (–1.22, 0.63)
Graduate		–1.5(–2.22,–0.80)	–0.1(–1.13,–0.88)			–0.9 (–1.16, 0.97)	–0.4 (–1.20, 0.33)			–0.5(–1.41,–0.34)

β: Regression estimate.

The results of multiple linear regression analysis are presented in Table 6. The analysis revealed that, DS and DT scores were associated with total CPQ_8-10_ and all the subscale scores (adjusted β range = 0.1 (95% CI: 0.03 to 0.19)–1.0 (95% CI: 0.72 to 1.26) and 0.2 (95% CI: 0.004 to 0.40)–1.2 (95% CI: 0.91 to 1.67), respectively). Untreated decay in primary and permanent dentition was associated with the total CPQ_8-10_ scores (adjusted β = 2.3 (95% CI: 0.67 to 3.91) and 3.4 (95% CI: 2.14 to 4.56), respectively) as well as the oral symptoms and functional limitations subscales (adjusted β range = 1.0 (95% C.I.: 0.32 to 1.64)–1.8 (95% CI: 0.43 to 3.10)). There was an association between oral hygiene index and total CPQ8-10 scores (adjusted β = 1.8 (95% CI: 0.62 to 3.02)), especially the functional limitations and emotional well-being subscales (adjusted β = 0.8 (95% CI: 0.16 to 1.44) and 0.4 (95% CI: 0.01 to 0.81), respectively).

**Table 6:** The effects of dental clinical measures on the child perception questionnaire (CPQ_8-10_) scores as assessed by multiple linear regression models.

	Total scores- CPQ_8-10_	Oral symptoms	Functional limitations	Emotional well-being	Social well-being
	β (95% CI)	β (95% CI)	β (95% CI)	β (95% CI)	β (95% CI)
DMFS	${1.0\;(0.71,\;1.26)}^{BSDP}$	${0.1(0.01,\;0.18)}^{SDP}$	${0.2\;(0.07,\;0.32)}^{BD}$	${0.2\;(0.13,\;0.26)}^{BSDP}$	${0.3\;(0.27,\;0.32)}^{BSP}$
DS	${1.0\;(0.72,\;1.26)}^{BSDP}$	${0.1\;(0.03,\;0.19)}^{SDP}$	${0.2\;(0.06,\;0.32)}^{BD}$	${0.2\;(0.13,\;0.26)}^{BSDP}$	${0.3\;(0.27,\;0.33)}^{BSP}$
MS	${\;1.3\;(–0.02,\;2.61)}^{BSDP}$	_	${0.5\;(–0.12,\;1.15)}^{BSD}$	${0.3\;(–0.13,\;0.76)}^{BSDP}$	${0.7\;(0.11,\;1.26)}^{BSDP}$
DMFT	${1.3\;(0.94,\;1.68)}^{BSDP}$	${\;0.2(0.009,\;0.38)}^{BSDP}$	${0.3\;(0.10,\;0.53)}^{BD}$	${0.3\;(0.15,\;0.45)}^{BSDP}$	${0.3\;(0.27,\;0.32)}^{BSP}$
DT	${1.2\;(0.91,\;1.67)}^{BSDP}$	${0.2\;(0.004,\;0.40)}^{SDP}$	${0.3\;(0.08,0.53)}^{BD}$	${0.3\;(0.14,\;0.45)}^{BSDP}$	${0.3\;(0.27,\;0.33)}^{BSP}$
MT	${\;6.4\;(–0.05,\;12.9)}^{BSDP}$	–	${2.8\;(–0.37,\;5.90)}^{BSD}$	${1.6\;(–0.68,\;3.82)}^{BSDP}$	${3.4\;(0.56,\;6.21)}^{BSDP}$
DT > 0	${3.4\;(2.14,\;4.56)}^{BSDP}$	${1.0\;(0.32,\;1.64)}^{BSDP}$	${1.0\;(0.39,\;1.68)}^{BSD}$	${0.6\;(0.17,\;1.02)}^{BSDP}$	${0.5\;(–0.05,\;1.01)}^{BSDP}$
dmfs	${0.3\;(0.17,\;0.42)}^{BSDP}$	${0.04\;(0.004,\;0.08)}^{EBSDP}$	${0.1\;(0.10,\;0.12)}^{BD}$	${0.04\;(0.01,\;0.07)}^{BSDP}$	${0.04\;(0.01,\;0.07)}^{BSDP}$
ds	${0.3\;(0.15,\;0.44)}^{BSDP}$	${\;0.04\;(–0.007,\;0.08)}^{EBSDP}$	${0.2\;(0.16,\;0.20)}^{BD}$	${0.1\;(0.01,\;0.17)}^{BSDP}$	${0.1\;(0.01,\;0.18)}^{BSDP}$
ms	${0.2\;(0.02,\;0.42)}^{BSDP}$	${\;0.04\;(–0.03,\;0.12)}^{BSDP}$	${0.08\;(0.001,0.16)}^{BSD}$	${0.03\;(–0.02,\;0.08)}^{BSDP}$	${0.01\;(–0.05,\;0.07)}^{BSDP}$
dmft	${0.6\;(0.34,\;0.86)}^{BSDP}$	${0.1\;(0.02,\;0.24)}^{EBSDP}$	${0.3\;(0.21,0.42)}^{BD}$	${0.1\;(0.01,\;0.19)}^{BSDP}$	${0.07\;(–0.02,\;0.\;16)}^{BSDP}$
dt	${0.6\;(0.33,\;0.78)}^{BSDP}$	${0.1\;(–0.008,\;0.23)}^{EBSDP}$	${0.3\;(0.21,\;0.44)}^{BD}$	${0.1\;(0.009,\;0.19)}^{BSDP}$	${0.08\;(–0.01,\;0.18)}^{BSDP}$
mt	${0.9\;(0.15,\;1.62)}^{BSDP}$	${0.2\;(–0.14,\;0.56)}^{EBSDP}$	${0.4\;(0.01,0.82)}^{BSD}$	${0.1\;(–0.12,\;0.36)}^{BSDP}$	${0.06\;(–0.25,\;0.38)}^{BSDP}$
dt > 0	${2.3\;(0.67,\;3.91)}^{BSDP}$	${1.8\;(0.43,\;3.10)}^{BSDP}$	${1.7\;(0.88,\;2.56)}^{BD}$	${0.01\;(–0.61,\;0.64)}^{BSDP}$	_
OHI	${1.8\;(0.62,\;3.02)}^{BSDP}$	${0.3\;(–0.35,0.92)}^{BSDP}$	${0.8\;(0.16,1.44)}^{BSD}$	${0.4\;(0.01,\;0.81)}^{BSDP}$	${0.4\;(–0.04,\;0.86)}^{BSDP}$
BI	${0.3\;(–0.01,\;0.64)}^{BSDP}$	${0.05\;(–0.14,\;0.24)}^{BSDP}$	${0.06\;(–0.14,\;0.27)}^{BSD}$	${0.03\;(–0.11,\;0.17)}^{BSDP}$	${0.2\;(0.04,\;0.36)}^{BSDP}$
CPI scores					
CPI = 1	${0.9\;(–0.39,\;2.2)}^{BSDP}$	${0.3\;(–0.28,\;0.92)}^{BSDP}$	–	–	${0.5\;(–0.01,\;1.01)}^{BSDP}$
CPI = 2	${1.7\;(–0.32,\;3.90)}^{BSDP}$	${0.5\;(–0.44,\;1.52)}^{BSDP}$	${1.2\;(0.16,\;2.24)}^{BSD}$	–	–

^E^ mother education

^A^ mother age

^F^ father education

^B^ brushing frequency

^S^ sweet snacks

^D^ dental visit

^P^ parent knowledge.

β: Regression estimate, DS/ds: Decayed surfaces, MS/ms: Missing surfaces, filled surfaces, DT/dt: Decayed teeth, MT/mt: Missing teeth, DT > 0/ dt > 0, prevalence of untreated decay, OHI-S: Simplified oral hygiene index, BI: Bleeding index, CPI: Community periodontal index, CPI = 1: Bleeding only, CPI = 2: With calculus.

**Fig 1:**
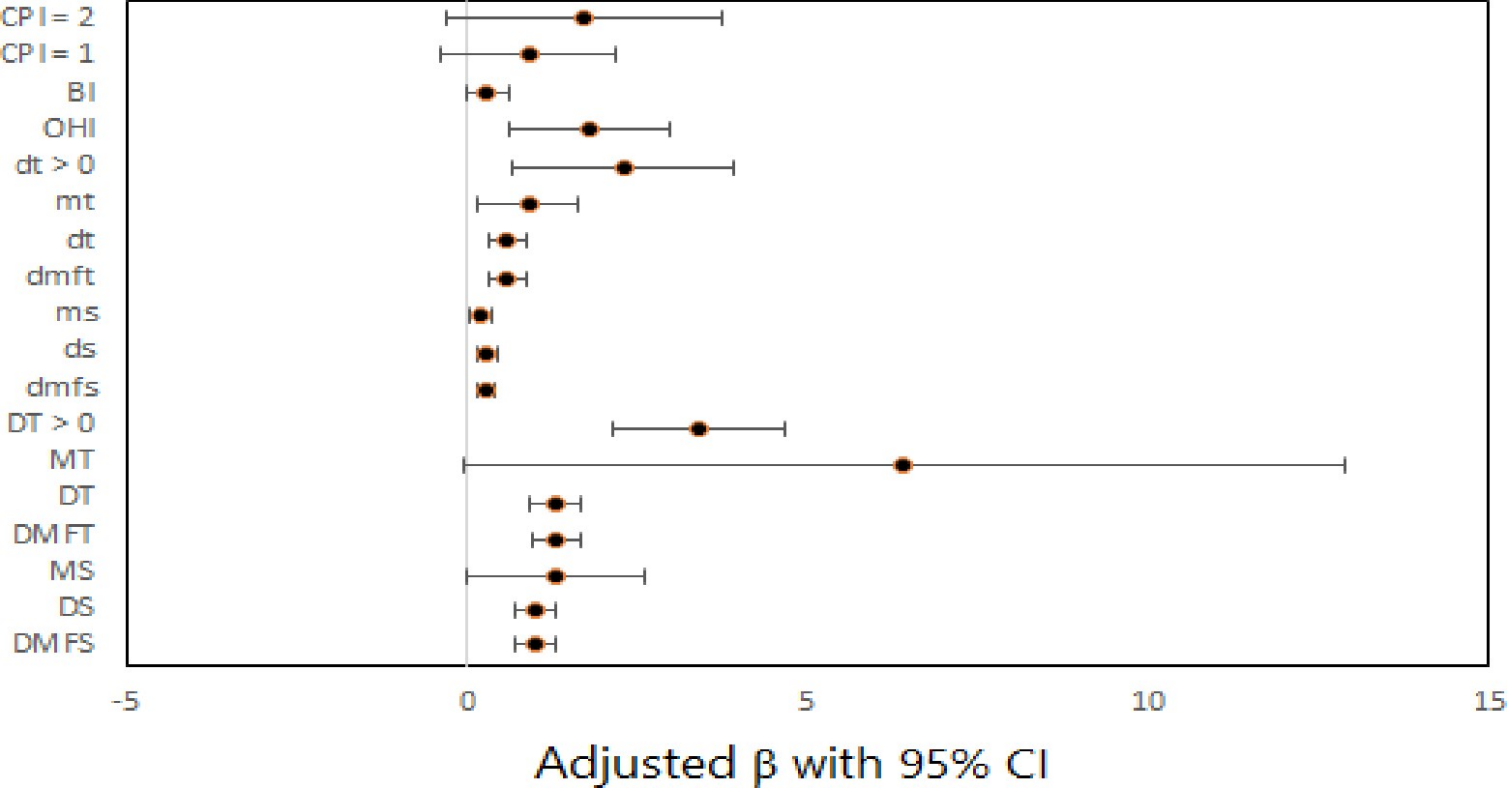
Forest plot for the effect of dental clinical measures on total CPQ_8-10_ scores by multiple linear regression models.

Fig 1 illustrates the effect of dental clinical measures on total CPQ_8-10_ scores after adjusting for covariates.

**Fig 2:**
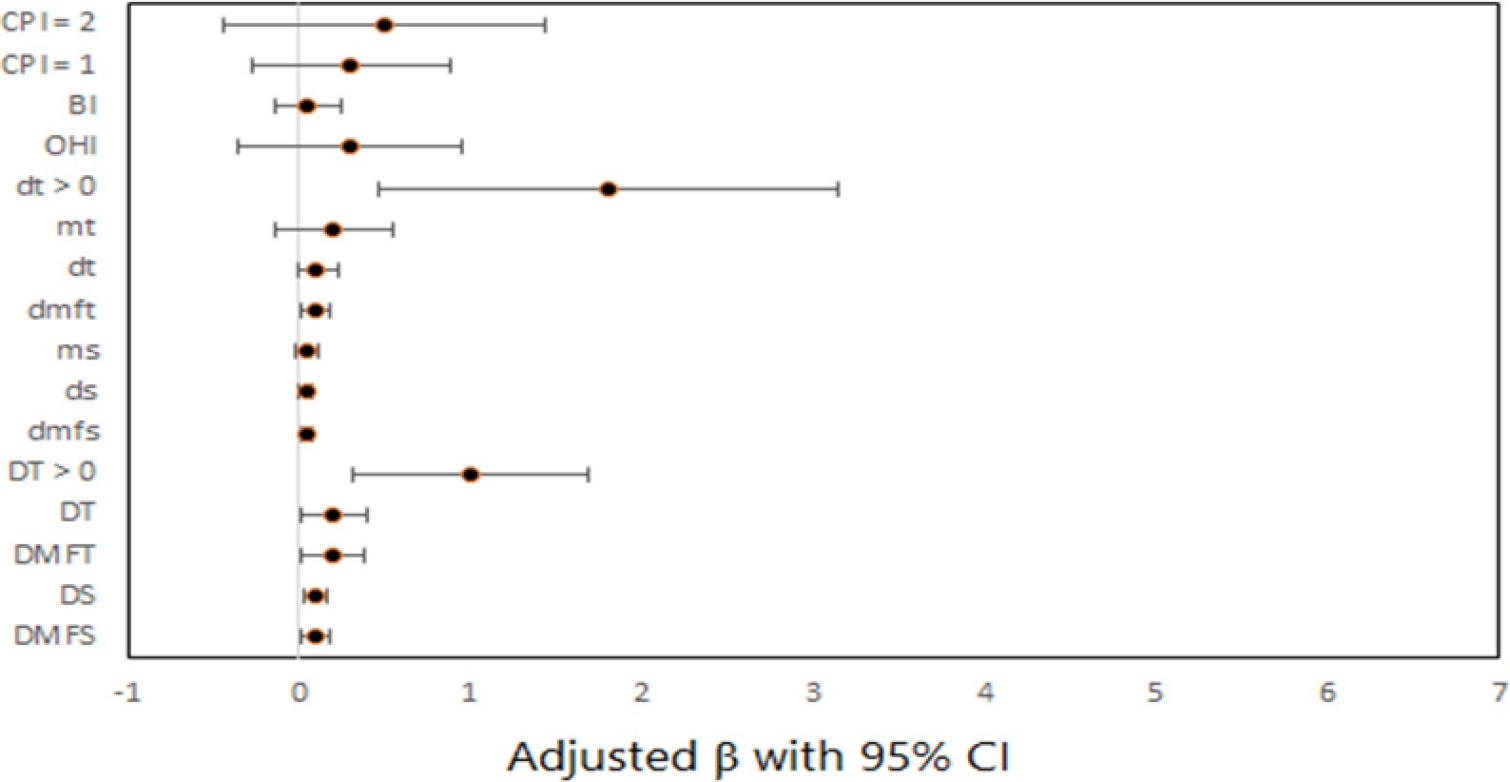
Forest plot for the effect of dental clinical measures on oral symptoms subscale score by multiple linear regression models.

The effect of dental clinical measures on oral symptoms subscale score after adjusting for covariates is presented in Fig 2.

**Fig 3:**
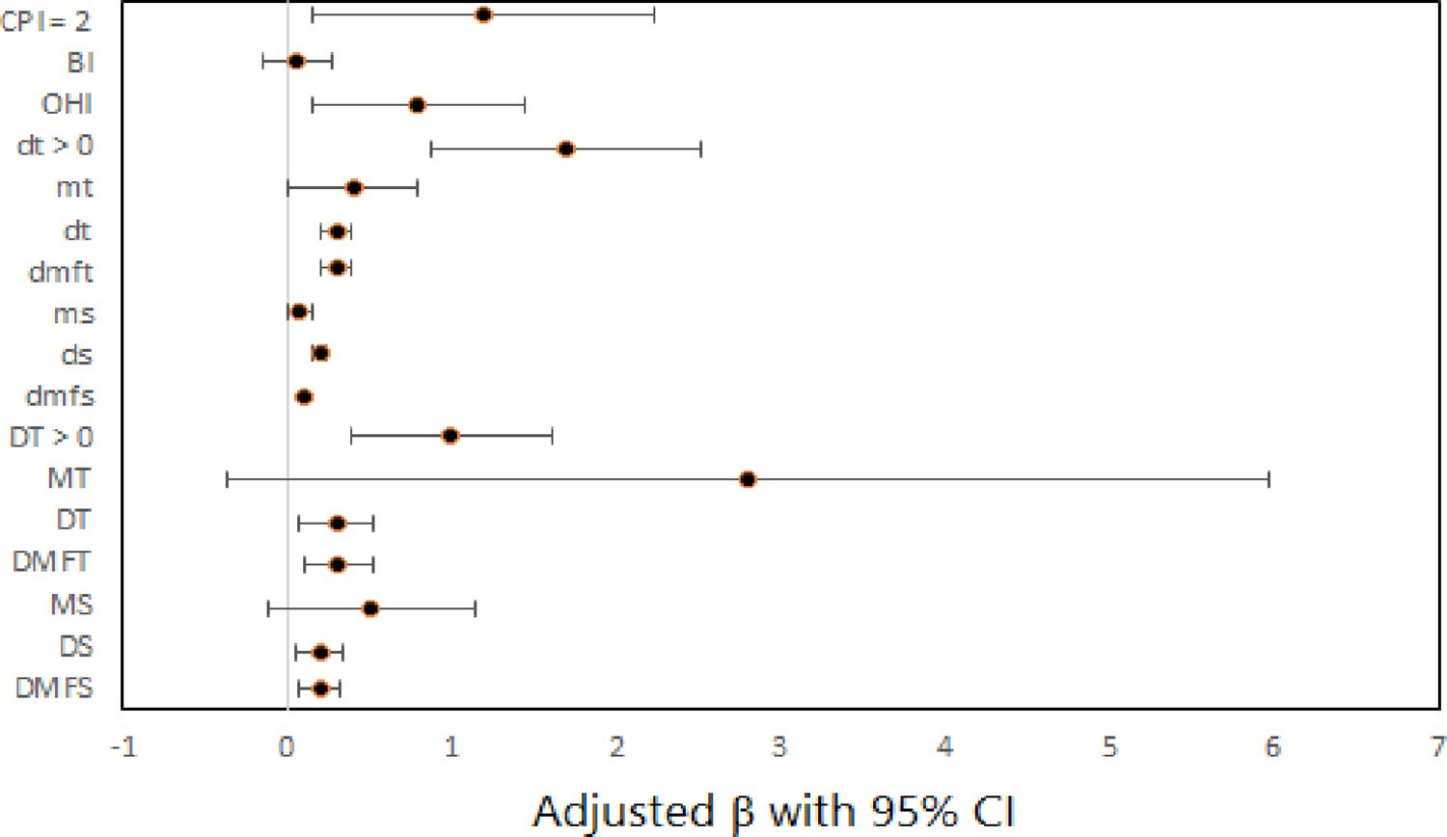
Forest plot for the effect of dental clinical measures on functional limitations subscale score by multiple linear regression models.

In addition, the effect of dental clinical measures on functional limitations subscale score after adjusting for covariates can be seen in Fig 3.

**Fig 4:**
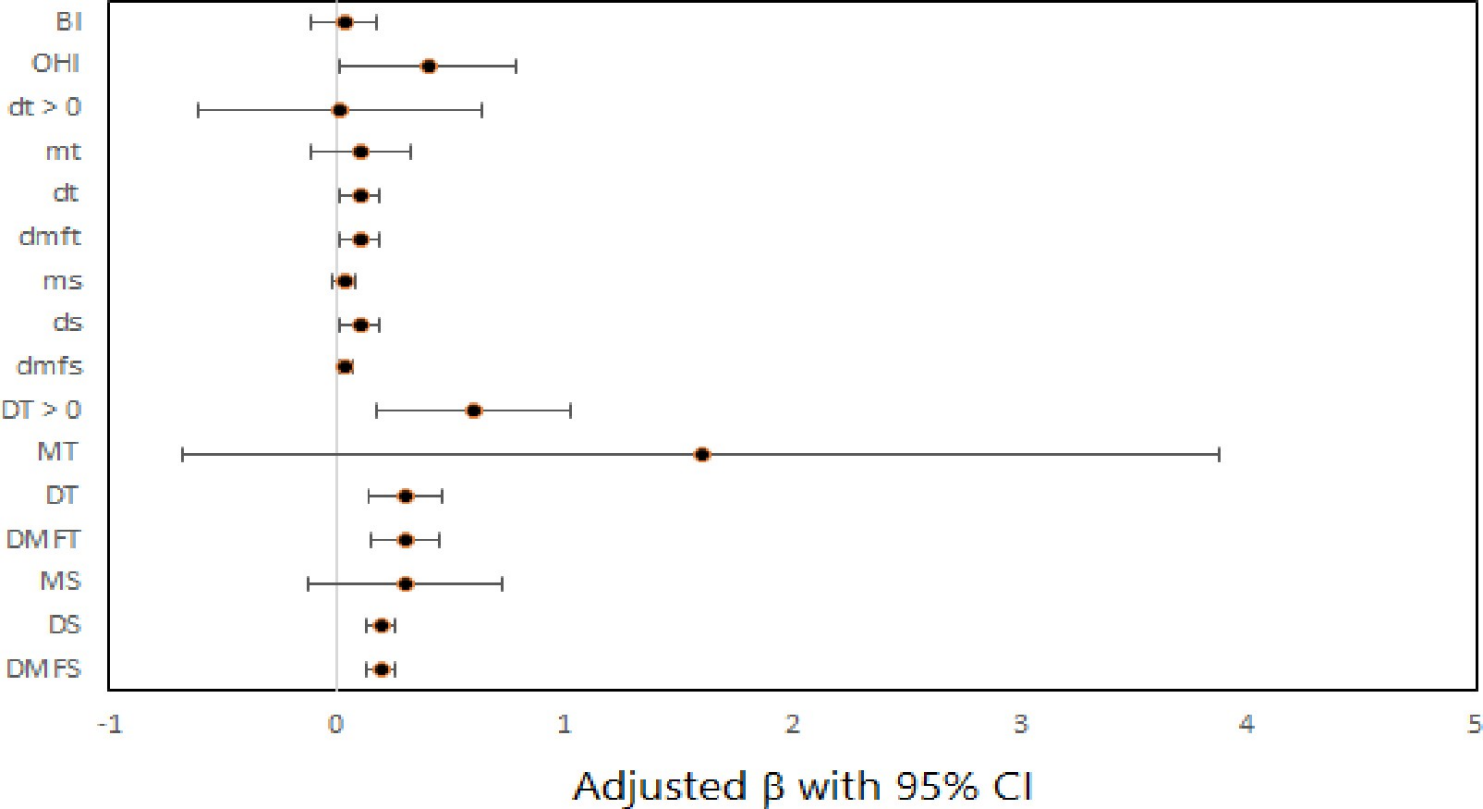
Forest plot for the effect of dental clinical measures on emotional well-being subscale score by multiple linear regression models.

Fig 4 demonstrates the effect of dental clinical measures on emotional well-being subscale score after adjusting for covariates.

**Fig 5:**
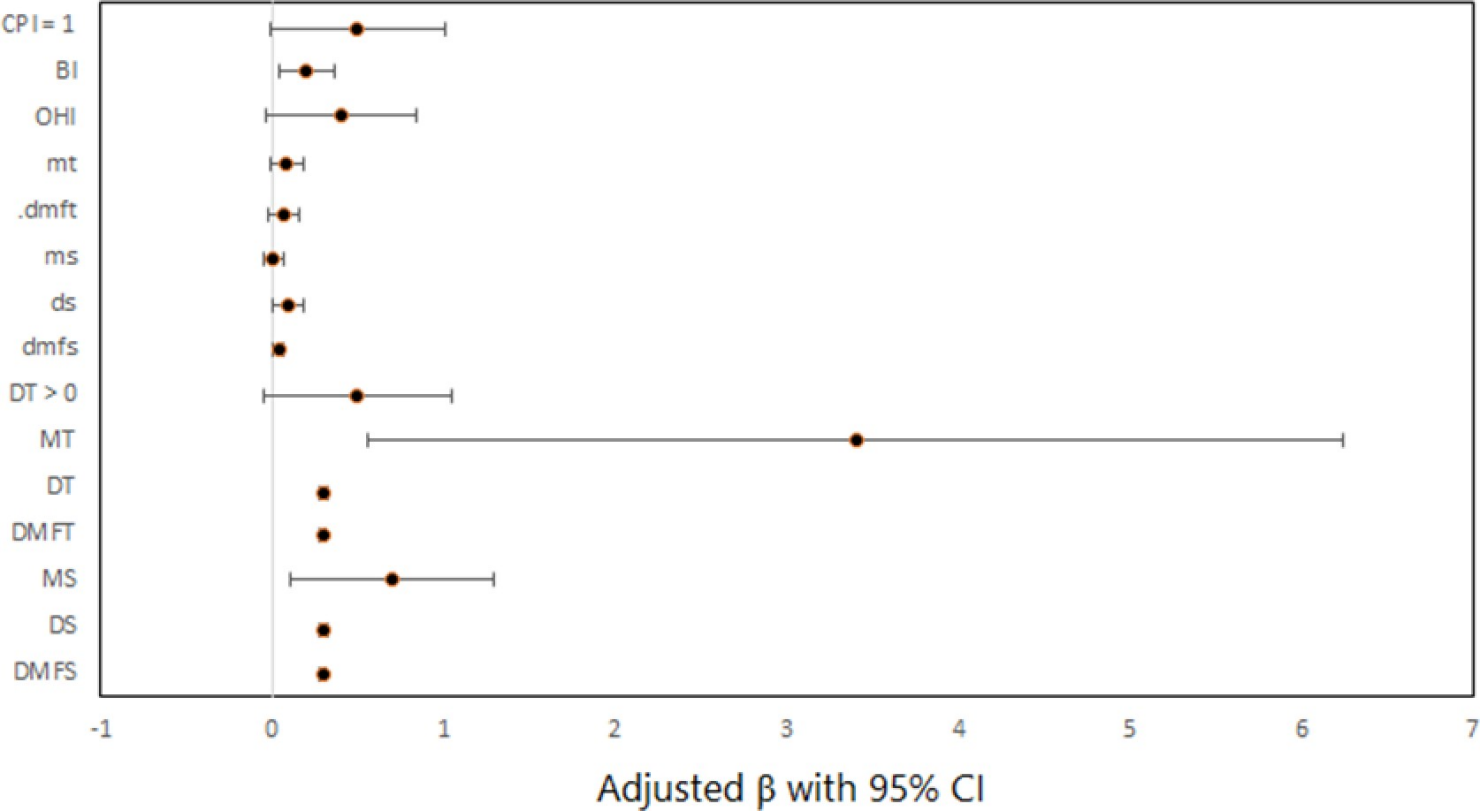
Forest plot for the effect of dental clinical measures on social well-being subscale score by multiple linear regression models.

Moreover, the effect of dental clinical measures on social well-being subscale score after adjusting for covariates is shown in Fig 5.

## Discussion

This study witnessed a relatively high prevalence of caries among the studied children in a way that at least three out of five children experienced caries either in primary or permanent dentition. This volume of the disease showed a clear impact on their quality of life as the higher the level of caries, the worse the status of OHRQoL. Among the factors which affect OHRQoL, children’s oral hygiene status, oral health behaviours and level of education of their mothers were more imperative.

The findings of this study contribute to the understanding of the association between dental caries with the OHRQoL of school-aged children. Schoolchildren with dental caries have reported a more negative effect on the overall subscales of the CPQ_8-10_, particularly in regards to the scores for the oral symptoms and functional limitations subscales. This finding aligns with another study conducted on the same age group and utilizing a similar assessment tool [33]. Additionally, Mota et al. found that the social well-being subscale had the highest prevalence ratio [34]. These studies provide support for the notion that the presence of dental caries has a detrimental impact on the quality of life. The observed difference in scores can be attributed to variations in data analysis methods. Specifically, after accounting for important covariates, it was determined that children with untreated dental caries had CPQ_8-10_ scores that were three units higher than those who were caries-free. In terms of domain-specific scores, the average CPQ_8-10_ score in this study was 14.7 (±7.64), with the oral symptoms domain exhibiting the highest mean scores. This discovery aligns with a similar study conducted in Brazil among 8- to 10-year-old schoolchildren [11]. Previous reports from Portugal and Saudi Arabia [24,35] have also indicated a correlation between poor oral hygiene status and lower OHRQoL scores, particularly in relation to functional limitations [36].

Our findings indicate that an increase of one unit in the oral hygiene index led to a corresponding increase of 1.8 units in the total scores of the CPQ_8-10_. These results further support the notion that maintaining good oral hygiene practices can positively influence the OHRQoL. A recent study conducted in Sri Lanka [13] reported no significant correlation between OHRQoL and the oral hygiene index, specifically the OHI-S. This disparity in findings may be partially attributed to differences in age. It is essential to acknowledge the potential influence of confounding factors due to the concurrent presence of other variables. There is a well-established association between sociodemographic factors and OHRQoL [20]. In our current study, we discovered that maternal education played a crucial role as a covariate, as children with educated mothers exhibited lower total CPQ_8-10_scores. This finding aligns with a recent study on Saudi children aged 12–14 years [24], which employed similar multivariate analysis techniques to identify the oral health measure that best predicted higher oral impact scores.

The results of our investigation demonstrated that an increase in the frequency of tooth brushing served as a protective factor for good OHRQoL. These findings align with similar observations made in Italy and New Zealand Italy and New Zealand [37,38]. Similarly, our results were in accordance with a recent study which indicated that the regular consumption of sugary items was found to be a risk factor for poor OHRQoL [13]. In the present study, the median score for parental knowledge regarding oral health and preventive measures for tooth decay was encouraging (33 out of a possible 40). Moreover, the proportion of children with parents who achieved a score equal to or greater than 33 was 58%, which is consistent with the results reported by Folayan et al. in their study on Nigerian children aged 8–12 years [25]. The current investigation demonstrated that the occurrence of dental caries in permanent dentition was 61%; this discovery is almost in accordance with previous investigations carried out in the Eastern Mediterranean Region [21]. Furthermore, the current investigation revealed that the occurrence of dental caries in primary dentition was 84%, which surpasses the rates documented from Libya [39] in a study that encompassed various age groups. A potential explanation for this observation is that parents underestimate the significance of deciduous teeth, ultimately resulting in either limited access to or inadequate utilization of dental care services.

In the present investigation, the average DMFT was 1.5 (±1.59), which is nearly comparable to a recent investigation in India [40], and in contrast to recent reports from Romania [41], and China [42]. The healthy CPI determined through this investigation was 38.7%, which closely aligns with the findings of a prior study carried out in Saudi Arabia [43], but contrasts with the results of a study conducted in Iran [44]. In general, discrepancies in the clinical assessment of oral health measures across various studies may be attributed to variations in geographical locations, age groups, demographics, and socio-economic characteristics of the study population. The present study demonstrates that girls exhibit higher levels of dental caries compared to boys. A similar gender disparity in dental caries was also observed in China [45]. This observation could potentially be explained by girls’ inclination towards cariogenic foods, coupled with the earlier eruption of teeth in girls relative to boys [46], consequently subjecting their teeth to a cariogenic oral environment for a longer duration than boys.

Our findings demonstrated that girls exhibited a higher occurrence of gingival bleeding compared to boys, which aligns with a study conducted on Chinese adolescent aged 12 years [47]. Prior research has substantiated that the prevalence of gingivitis during puberty is associated with an elevation in systemic levels of sex hormones [48,49]. Furthermore, the onset of gingivitis in girls tends to occur at an earlier age than in boys [49,50]. Consequently, it is plausible to suggest that the increased levels of sex hormones in girls may exert an influence on these observed outcomes.

This investigation possesses specific advantages, such as its focus on examining the impact of dental caries on OHRQoL of 8-10-year-old children, particularly within the context of a developing nation like Iraq. To determine the consistent oral health measures that influence OHRQoL, the present study employed the use of the relative effect size (regression estimate; β) and the precision of the confidence intervals derived from multiple linear regressions. These regressions were adjusted for various covariates, including sociodemographic factors and oral health behaviour, in order to provide a more precise assessment of the impact. Another strength of this investigation may be attributed to the fact that the findings were obtained from a homogeneous population, and the analysis took into account significant demographic variables and oral health-related behaviours. Furthermore, the assessment of OHRQoL was conducted using a locally validated tool known as the Child Perceptions Questionnaire for 8-10-year-olds [29].

On the other hand, one potential limitation that may be applicable to this study is the utilization of a cross-sectional study design, which hinders the establishment of a causal relationship between dental clinical measures and OHRQoL. Additionally, there exists a potential risk of social desirability bias, especially when self-administered questionnaires completed by parents are employed as proxies for their children [51].

## Conclusion

The results of this study give considerable importance to the detrimental influence of dental caries and inadequate oral hygiene on the overall OHRQoL of the studied children. This particular association becomes even more pronounced when taking into account the fact that more than two-thirds of these children fail to meet the desirable standards of oral health behaviour. In order to improve the level of OHRQoL among this specific group of children, it is imperative to implement comprehensive initiatives that aim to reduce the prevalence of unmet oral health needs, while simultaneously promoting adherence to recommended oral health practices. These initiatives should encompass a wide range of strategies and interventions, covering both preventive and curative measures, in order to achieve optimal outcomes for these children. The current findings provide a significant contribution to the decision-making process in clinical settings and the establishment of priorities within public oral health care. In Iraq, future oral health policies should focus on preventive measures against caries and its associated complications among schoolchildren.

## Supporting information

S1 Database (372 participants). Also available via doi: 10.5061/dryad.9ghx3ffpw. (XLSX)
